# Cytopenia associated with copper deficiency

**DOI:** 10.1002/jha2.278

**Published:** 2021-08-26

**Authors:** Kaori Uchino, Lam Vu Quang, Megumi Enomoto, Yuta Nakano, Saki Yamada, Saori Matsumura, Jo Kanasugi, Soichi Takasugi, Ayano Nakamura, Tomohiro Horio, Satsuki Murakami, Mineaki Goto, Shohei Mizuno, Hidesuke Yamamoto, Masaya Watarai, Ichiro Hanamura, Akiyoshi Takami

**Affiliations:** ^1^ Division of Hematology, Department of Internal Medicine Aichi Medical University School of Medicine Nagakute Japan; ^2^ Hematopoietic Cell Transplantation Center Aichi Medical University Hospital Nagakute Japan; ^3^ Department of Clinical Laboratory Aichi Medical University Hospital Nagakute Japan; ^4^ Keyaki Clinic Hashima‐gun Gifu Japan; ^5^ Division of Hematology/chemotherapy Daido Hospital Nagoya Japan

**Keywords:** copper deficiency, cytopenia, cytoplasmic vacuoles, zinc supplements

## Abstract

**Introduction:**

Due to an increased incidence of copper deficiency, we investigated adult patients who had low serum levels of copper with cytopenia at our hospital from March 2014 to March 2021.

**Methods:**

We retrospectively reviewed the clinical data of patients who had been diagnosed with cytopenia due to copper deficiency at the Aichi Medical University Hospital from March 2014 to March 2021.

**Results:**

In the 15 patients with cytopenia secondary to low serum copper level, 11 had cytopenia of two to three lineages; three (27%) had pancytopenia, and eight (73%) had bicytopenia. Of the 15 patients, nine (60%) underwent bone marrow examinations; three (30%) showed typical morphologic features associated with copper deficiency, such as multiple clear cytoplasmic vacuoles in erythroblasts and myeloid cells, and three (30%) showed dysplastic features as observed in myelodysplastic syndrome. Among the 14 (93%) patients who were treated with copper supplements, had cessation of zinc supplements, or both, 11 (73%) and eight (53%) showed normal copper levels and hematological improvement, respectively.

**Conclusion:**

Copper deficiency is more common than expected and should be considered in patients with unexplained cytopenia.

## 1 INTRODUCTION

Copper is an essential trace element that functions as an enzymatic cofactor in processes involving connective tissue formation, iron metabolism and hematopoiesis, and central nervous system function [1]. The adult human body contains only about 50–120 mg of copper, which is very low compared to other trace elements such as iron and zinc [2]. The recommended safe and adequate dietary intake of copper for adults is 700–900 μg /day [3]. Copper is absorbed primarily in the small intestine, with a small amount absorbed in the stomach, transported via the portal blood to the liver, and incorporated into ceruloplasmin. Ceruloplasmin is released into the blood and delivers copper to tissues throughout the body [2].

Copper deficiency is less common in healthy individuals because copper is a ubiquitous element and is present in almost all foods [4]. In the past, copper deficiency has been observed in preterm infants and patients with hereditary conditions, such as Menkes disease [5]. Increased use of enteral nutrition (EN) and total parental nutrition (TPN), advances in medical techniques, and an increase in the aging population has led to increased copper deficiency [6]. Moreover, copper deficiency induced by excessive zinc has also increased because zinc supplements have been common.

Copper deficiency causes hematological abnormalities such as anemia, leukopenia, neutropenia, and thrombocytopenia. However, diagnosis of cytopenia due to copper deficiency is difficult because the bone marrow morphology in copper deficiency cannot be distinguished from myelodysplastic syndrome (MDS) [2, 7, 8, 9]. This difficulty prompted us to retrospectively investigate and analyze the clinical and hematologic features of patients with copper deficiency.

## 2 PATIENTS AND METHODS

### 2.1 Patients

We obtained ethical approval from the Institutional Review Board of Aichi Medical University School of Medicine. Patients diagnosed with cytopenia due to copper deficiency at the Aichi Medical University Hospital from March 2014 to March 2021 were enrolled in the study. We defined copper deficiency as serum copper level below the lower limit of normal (< 68 μg/dL). Leukopenia, neutropenia, anemia, and thrombocytopenia were defined as white blood cells, neutrophils, red blood cells, and platelet counts < 3000 /μL, < 1500/μL, < 11 g/dl, and < 10,000 /μL, respectively.

We retrospectively collected data on the clinical history, hematologic examination findings, and treatments of copper deficiency. Complete blood count and bone marrow morphology were reviewed, and their relationship with the copper concentration was investigated. Moreover, we divided the patients into two groups according to the median value of serum copper level (<16 and ≥16 μg/dL) and compared the hematological features and therapeutic effect between the two groups.

### 2.2 Data management and statistical analyses

The student's *t*‐test was applied to compare the means of continuous variables and normal distribution data and was carried out using the EZR software package [10].

## 3 RESULTS

### 3.1 Patients' characteristics

A total of 15 patients (ten men and five women) with cytopenia secondary to low serum copper level were identified. The median (range) age of the participants was 69 (33–88) years. In patients with low copper level, eight (53%) had a prescription of zinc supplements, four (27%) had EN/TPN, one (6.7%) had a feeding disorder, and one (6.7%) had malabsorption. In addition, among the patients who received zinc supplements, five (33%) underwent hemodialysis, and two (13%) had liver cirrhosis. Zinc acetate dihydrate (ZAH: Nobelzin®, Nobelpharma Co., Ltd) was the most commonly administered zinc supplement and polaprezinc (PPZ: Promac®, Zeria Pharmaceutical Co., Ltd.), which contains less zinc than ZAH, was the second most administered zinc supplement. The clinical and hematological characteristics of the patients are summarized in Tables 1 and 2.

**TABLE 1:** Patient characteristics (*n* = 15)

Variable	Value
Patient age, years, median (range)	69 (33–88)
Sex, *n* (%)	
Male	10 (67)
Female	5 (33)
Underlying problem, *n* (%)	
Prescription of zinc supplements	8 (53)
Polaprezinc	5 (33)
Zinc acetate dihydrate	3 (20)
Enteral nutrition or total parenteral nutrition	4 (27)
Long‐term eating disorder	1 (6.7)
Malabsorption	1 (6.7)
Unknown	1 (6.7)

**TABLE 2:** Characteristics of patients with copper deficiency

No.	Age	Sex	Leukocytes (/μL)	Neutrophils (/μL)	Hemoglobin (g/dL)	MCV (fL)	Reticulocytes (/μL)	Platelets (10^4^/μL)	Serum copper (μg/dL)	Serum zinc (μg/dL)	Vitamin B_12_ (pg/mL)	Folic acid (ng/mL)	Ferritin (ng/mL)	Underlying disease	BMA findings	G‐banding analysis of bone marrow	Cause of copper deficiency	Treatment	Response to treatment
1	69	Female	5400	4660	7.2	96.4	N/A	7.6	35	18	312	4.5	1060	Chronic inflammation	N/A	N/A	Long‐term eating disorder	Copper supplementation	Normalization of serum copper level and partial hematologic response
2	33	Male	4300	3225	8.9	102	94320	27	14	45	420	10.3	48.8	Malabsorption	Dysplastic features	46, XY [20]	Malabsorption	Copper supplementation	Normalization of serum copper level and no hematologic response
3	72	Male	2800	952	6.8	116	38900	16.1	0	59	1310	22	527.4	After cerebral infarction	Cytoplasmic vacuoles	46, XY [20]	Enteral nutrition or total parenteral nutrition	Copper supplementation	Normalization of serum copper level and complete hematologic response
4	72	Male	1700	476	6.7	106.4	40600	5.1	0	128	762	9.5	1323	Multiple myeloma	Dysplastic features	46, XY [20]	Prescription of zinc supplements (zinc acetate dihydrate)	Copper supplementation and cessation of zinc supplements	Normalization of serum copper level and partial hematologic response
5	59	Male	2200	1166	8.7	102	30000	11.1	6	135	1500	2.3	N/A	Diabetic neuropathy	Cytoplasmic vacuoles	45, XY, der(13;14) (q10;q10) [20]	Prescription of zinc supplements (polaprezinc)	Copper supplementation	Normalization of serum copper level and complete hematologic response
6	60	Male	1900	1672	7.6	90.2	10200	2	12	112	N/A	N/A	1804	After chemoradiotherapy	No abnormality	46, XY [20]	Prescription of zinc supplements (zinc acetate dihydrate)	Copper supplementation and cessation of zinc supplements	Normalization of serum copper level and complete hematologic response
7	88	Female	2150	1391	11	101	44600	7.5	17	N/A	N/A	N/A	85	Myelodysplastic syndrome	Cytoplasmic vacuoles	46, XX [20]	Prescription of zinc supplements (polaprezinc)	Copper supplementation	Normalization of serum copper level and no hematologic response
8	75	Male	2700	1695	9	91.2	89958	11.9	11	221	N/A	N/A	14.5	Liver cirrhosis	No abnormality	45, X, ‐Y [12]/ 44, idem, ?t(7;11)(p21;q12), ‐21 [1]/ 46, XY [7]	Prescription of zinc supplements (zinc acetate dihydrate)	Copper supplementation and cessation of zinc supplements	Normalization of serum copper level and partial hematologic response
9	67	Female	5700	4902	8.5	92.1	16800	2.8	18	N/A	1100	6.7	784	Chronic inflammation	Dysplastic features	46, XX [20]	Enteral nutrition or total parenteral nutrition	Copper supplementation	Not achieved normalization of serum copper level or partial hematologic response
10	61	Male	16200	15221	7.8	94.4	52300	5.1	19	43	441	33.8	617	Chronic inflammation	N/A	N/A	Enteral nutrition or total parenteral nutrition	No treatment	N/A
11	52	Male	4000	3640	6.9	88.4	N/A	4.1	16	98	1500	5.5	2747	Chronic inflammation	N/A	N/A	Enteral nutrition or total parenteral nutrition	Copper supplementation	Normalization of serum copper level and partial hematologic response
12	85	Male	4100	2255	6.9	102	43100	13.5	10	143	529	5.1	678	Hemolytic anemia	No abnormality	45, X, −Y [5] / 46, XY [15]	Prescription of zinc supplements (zinc acetate dihydrate)	Copper supplementation and cessation of zinc supplements	Normalization of serum copper level and complete hematologic response
13	53	Male	4600	2530	5.9	101.7	41900	12.6	16	64	991	22	57.3	Liver cirrhosis	N/A	N/A	Prescription of zinc supplements (zinc acetate dihydrate)	Cessation of zinc supplements	Normalization of serum copper level and no hematologic response
14	76	Female	5400	4698	8.9	144.2	77600	30.3	29	N/A	N/A	N/A	N/A	Acquired pure red cell aplasia	N/A	N/A	Prescription of zinc supplements (polaprezinc)	Copper supplementation and cessation of zinc supplements	Not achieved normalization of serum copper level or partial hematologic response
15	78	Female	3100	2108	8.1	91.5	71900	1.9	28	45	321	3.3	3076	Aplastic anemia	N/A	N/A	Unknown	Copper supplementation	Not achieved normalization of serum copper level or partial hematologic response

Abbreviations: MCV, mean corpuscular volume; BMA, bone marrow aspiration; N/A, not applicable.

### 3.2 Hematological and laboratory features

Cytopenia in two to three lineages was observed in 11 (73%) patients, with pancytopenia in three (20%), anemia and leukopenia in three (27%), anemia and thrombopenia in four (27%), and leukopenia and thrombopenia in one (6.7%) patient (Table 3); four patients (27%) had isolated anemia. Neutropenia was seen in four patients (27%). Seven patients (47%) with anemia developed normochromic anemia and eight (53%) developed macrocytic anemia. Vitamin B12 and folic acid levels were measured in 11 patients (73%) and were within the normal range in nine patients. Two patients with low folic acid level did not improve their cytopenia after increasing their levels of folic acid.

**TABLE 3:** Hematologic and laboratory features of patients with copper deficiency

Variable			Number of cases (%)
Pancytopenia			3 (20)
Anemia and leukopenia			3 (20)
Anemia and thrombopenia			4 (27)
Leukopenia and thrombopenia			1 (6.7)
Isolated anemia			4 (27)
Isolated leukopenia			0 (0)
Isolated thrombopenia			0 (0)
Neutropenia			4 (27)
Bone marrow aspiration			9 (60)
Dysplastic features			3 (20)
Cytoplasmic vacuoles			3 (20)
Hemosiderin containing plasma cells			1 (6.7)
Megakaryocyte with the markedly abnormal nucleus			1 (6.7)
No abnormalities			3 (20)

	Reference ranges	Median	Range
Hemoglobin (g/dL)	11.4–14.8	7.8	5.9–11
MCV (fL)	81–97	101	88.4–144.2
Reticulocytes (/μL)	14000–92000	43100	10200–94320
Leukocytes (/μL)	5000–8000	4000	1700–16200
Neutrophils (/μL)	2130–4712	2255	476–15221
Platelets (10^4^/μL)	18–35	7.6	1.9–30.3
Serum copper (μg/dL)	68–128	16	0–35
Serum zinc (μg/dL)	80–130	81	18–221
Vitamin B_12_ (pg/mL)	180–914	762	312–1500
Folic acid (ng/mL)	>4.0	6.7	2.3–33.8
Ferritin (ng/mL)	5.0–179	678	14.5–3076

Abbreviation: MCV, mean corpuscular volume.

Eleven (73%) patients suffered from conditions that may have influenced the hematologic presentation, including chronic inflammation‐like infection (*n* = 4, 27%), liver cirrhosis (*n* = 2, 13%), MDS (*n* = 1, 6.7%), aplastic anemia (*n* = 1, 6.7%), hemolytic anemia (*n* = 1, 6.7%), acquired pure red cell aplasia (*n* = 1, 6.7%), multiple myeloma (*n* = 1, 6.7%), and after chemoradiotherapy (*n* = 1, 6.7%).

Among the patients with copper deficiency, three patients (20%) had higher serum zinc levels than 130 μg/dL, which is the upper limit of the normal range in our laboratory.

**FIGURE 1:**
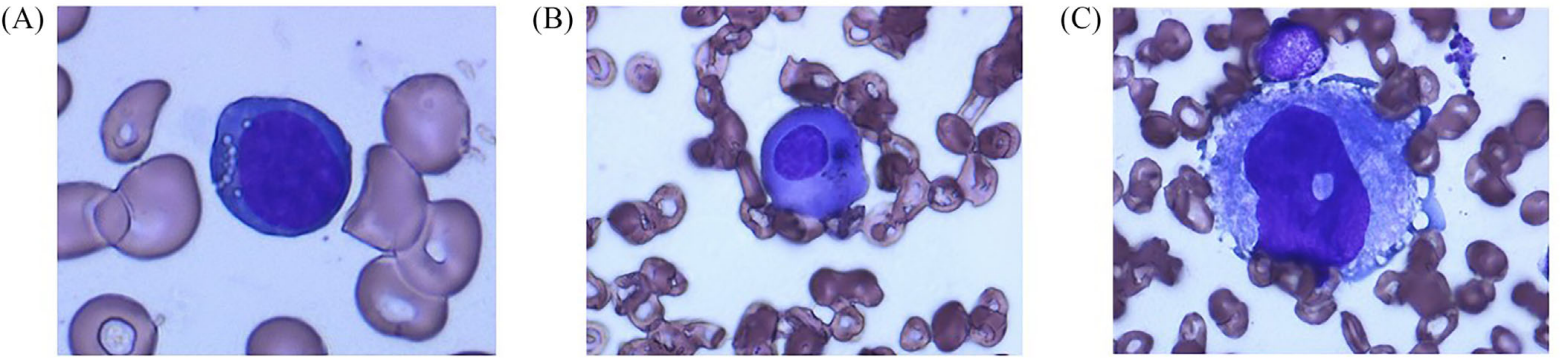
Typical morphologic features associated with copper deficiency: (A) cytoplasmic vacuoles in erythroid precursor, (B) hemosiderin containing plasma cells, and (C) megakaryocyte with the markedly abnormal nucleus

Nine (60%) patients underwent a bone marrow examination, in which three patients (20%) showed typical morphologic features associated with copper deficiency, such as multiple clear cytoplasmic vacuoles in erythroblasts and myeloid cells. Hemosiderin‐containing plasma cells and megakaryocytes with markedly abnormal nuclei were noted in one patient (Figure 1). Three patients (20%) showed dysplastic features, as observed in the MDS.

We divided the patients into two groups according to the median value of serum copper level (< 16 and ≥16 μg/dL) and compared the hematological features and therapeutic effect between the two groups. The < 16 μg/dL serum copper group showed a trend toward a lower neutrophil count (*p* = 0.076). There was no statistical difference in the other hematologic parameters or time from diagnosis to normalization of serum copper level (Table 4).

**TABLE 4:** Association between hematological features and serum copper level

Variable	Serum copper (μg/dL)		
	<16	≥16	*p*
Number of cases			
Hemoglobin (g/dL)	7.6	7.95	0.74
MCV (fL)	102	95.4	0.98
Leukocytes (/μL)	2700	5000	0.10
Neutrophils (/μL)	1672	4150	0.076
Platelets (/μL)	11.9	6.3	0.47
Time from diagnosis to normalization of serum copper level (days)	91	67	0.37

Abbreviation: MCV, mean corpuscular volume.

### 3.3 Treatment and responses to therapy

Fourteen patients (93%) were treated with copper supplements, cessation of zinc supplements, or both. We administered pure cocoa powder as a copper supplement, which is the richest source of dietary copper and contains approximately 4 mg/100 g weight. Some patients could not take the cocoa powder and chose other foods such as nuts, shellfish, liver, or chocolate as sources of dietary copper. Intravenous administration of combined manganese chloride zinc sulfate hydrate (Volvix®, Yakult Honsha Co., Ltd) and oral nutrition supplements were also used as copper supplements. The serum copper level normalized in 11 (73%) patients (Table 5). Three patients (20%) did not improve their serum copper levels because the amount of copper they took was less than the amount of adequate dietary intake of copper for adults. The cytopenia improved in eight (53%) patients who had serum copper evaluation; four patients (27%) had complete normalization of their hematologic indices. Four patients (27%) showed a partial response to therapy. Patients who had no improvements following copper supplements were later diagnosed with MDS or liver cirrhosis. The patient with malabsorption showed persistent anemia, although his serum copper level increased to normal. Only one patient died before the initiation of copper supplements.

**TABLE 5:** Treatment and responses to therapy

Variable	Number of cases (%)
Types of therapy	
Copper supplementation	8 (53)
Cessation of zinc supplements	1 (6.7)
Both copper supplementation and cessation of zinc supplement	5 (33)
No treatment	1 (6.7)
Response to therapy	
Normalization of serum copper level	11 (73)
Complete hematologic response	4 (27)
Partial hematologic response	4 (27)
No hematologic response	2 (13)
Unevaluable	1 (6.7)

	Median duration (range)
Time from diagnosis to normalization of serum copper level (days)	84 (28–461)

## 4 DISCUSSION

The present study showed the results of the analysis of 15 patients with cytopenia secondary to copper deficiency. Zinc supplementation and bicytopenia were most commonly associated with copper deficiency. Low serum copper level (<16 μg/dL) was also associated with a lower neutrophil count. Not all patients had typical morphologic bone marrow features associated with copper deficiency, and in some patients dysplastic features seen in MDS were observed.

The mechanisms of copper deficiency‐induced cytopenia have not yet been fully investigated. Anemia and neutropenia are common, whereas thrombocytopenia is reported to be uncommon in copper deficiency [6]. Copper is an important component of ceruloplasmin and hephaestin, which are necessary for the export and absorption of iron and synthesis of heme [11, 12]. Moreover, copper deficiency has been shown to reduce the lifespan of red cells [13]. We attempted to verify the relationship between copper and iron metabolism in our study. Although some patients showed low total iron‐binding capacity, unsaturated iron‐binding capacity, and high ferritin levels, it was difficult to determine whether copper deficiency induced iron unavailability because these patients had chronic inflammation and underwent repeated red blood cell transfusion, which increases ferritin level.

Some studies have also suggested that copper deficiency arrests neutrophil mutation and leads to the development of anti‐antibodies resulting in neutropenia [14, 15]. Our finding that some patients with copper deficiency developed leukopenia and neutropenia supports these previous reports. Moreover, we found that severe low copper deficiency frequently led to much lower neutropenia than mild low copper deficiency. To our knowledge, this is the first report on the relationship between neutrophil count and the extent of decrease in serum copper levels.

Although isolated anemia, bicytopenia, and pancytopenia have been reported, there is no report about isolated thrombocytopenia because of copper deficiency and its mechanisms. In our study, five patients (33%) with thrombocytopenia coexisted with anemia and leukopenia and improved their platelet count after treatment. Therefore, we suspected that thrombocytopenia caused by copper deficiency might have occurred in the case of severe and long‐term copper deficiency. However, there was no significant difference between the platelet count and the extent of decrease in serum copper level. Moreover, it makes the analysis difficult because the details regarding the duration of copper deficiency were not within the scope of the current retrospective study, and thrombocytopenia is caused by copper deficiency and various conditions. Therefore, further studies with a larger sample size are necessary to investigate our hypothesis and establish the mechanisms of thrombocytopenia caused by copper deficiency.

The results of our study suggest that the most common cause of copper deficiency was zinc supplementation in this study. Reports of copper deficiency due to zinc supplements have increased since the 2000s, and the mechanism that zinc induces copper deficiency suggested [16, 17, 18, 19, 20, 21, 22, 23, 24, 25]. Excessive zinc stimulates enterocytes to produce metallothionein, and copper has a higher affinity for metallothionein than zinc. Therefore, copper binds to metallothionein and then sloughs into the stool, resulting in copper deficiency [26]. Zinc deficiency is developed frequently in patients on hemodialysis or who have liver cirrhosis [27, 28, 29, 30]. In the present study, we also experienced that the patients who were on maintenance hemodialysis or had liver cirrhosis developed copper deficiency, possibly due to zinc supplements. Although our patient developed anemia 8 months after she started PPZ, there was a report that cytopenia due to copper deficiency with PPZ developed earlier than in our patient [31]. Moreover, patients prescribed ZAH, which contains more zinc than PPZ, were reported to develop copper deficiency more frequently and earlier than patients prescribed PPZ [32]. This report is compatible with our result that more patients prescribed with ZAH experienced cytopenia due to copper deficiency than patients prescribed with PPZ. Therefore, in patients with cytopenia, it is important to elicit history zinc supplements and consider the risk of copper deficiency induced by excessive zinc, especially in patients undergoing hemodialysis and liver cirrhosis.

Our study also suggests that the second most common cause of copper deficiency was EN or TPN in this study. Total copper intake from the 900–1500 kcal/day tube feeding formula, which contains 5–12 μg of copper per 100 kcal, is approximately 50–180 μg/day [33]. Some TPN fluids include only approximately 300 μg of copper per 2000 kcal, while others do not contain any copper. These amounts of copper are less than the recommended safe and adequate dietary intake of copper for adults (700–900 μg/day) [3]. Prolonged EN or TPN may cause copper deficiency. Pancytopenia due to copper deficiency was reported to develop only 8 weeks after the removal of copper from TPN [34]. It is important to regularly check serum copper levels in patients who receive EN or TPN.

The treatment for copper deficiency is not yet developed. However, it is easy to consume food containing high amounts of copper as a copper supplement. The amount of copper included in the intravenous administration of manganese chloride zinc sulfate hydrate combined with oral nutrition supplements was less than in copper‐rich food. The patient in our study who received only intravenous administration of manganese chloride zinc sulfate hydrate combined with oral nutrition supplements did not increase his serum copper level. Commercially available pure cocoa contains approximately 4 mg copper per 100 g and is easy to administer to tube‐feeding patients because it is in powder form. We recommended other copper‐rich foods such as nuts, shellfish, or liver to patients with diabetes mellitus because almost all patients add sugar to suppress the bitterness of cocoa. We also used other copper‐rich foods for patients who could not continue oral cocoa intake due to diarrhea because cocoa contains fibers [35]. We suggest that copper supplements should be chosen depending on the patient's underlying conditions.

This study has some limitations. One major limitation is the small number of patients included in this study. In addition, detailed information on the clinical presentation induced by copper deficiency other than cytopenia, such as neurologic symptoms, duration of copper deficiency, laboratory parameters, and bone marrow examination, was not available for all patients because this study was retrospective.

In conclusion, most cytopenia secondary to copper deficiency occurred in patients receiving zinc supplements, EN, or TPN. The number of patients with impaired hematopoiesis due to copper deficiency is expected to increase because the number of patients undergoing dialysis, EN, or TPN is also expected to increase with the advancement of medicine. Diagnosis of cytopenia due to copper deficiency is difficult because these patients have many problems that can cause impaired hematopoiesis. Additionally, the bone marrow morphologies in copper deficiency, which showed dysplastic features similar in MDS, complicate the diagnosis. Therefore, in cytopenia patients with zinc supplements or prolonged EN or TPN, copper deficiency should be considered in the differential diagnosis.

## FUNDING STATEMENT

The funders played no role in the study design, data collection, and analysis, the decision to publish or the preparation of the manuscript.

## CONFLICT OF INTEREST

The authors declare no conflict of interest in association with the present study.

## AUTHOR CONTRIBUTIONS

K. Uchino and A. Takami designed the study. K. Uchino performed the statistical analysis wrote the paper. L.V. Quang, M. Enomoto, Y. Nakano, S. Yamada, S. Matsumura, J. Kanasugi, S. Takasugi, A. Nakamura, T. Horio, S. Murakami, M. Goto, S. Mizuno, H. Yamamoto, M. Watarai, and I. Hanamura contributed to data collection. All authors have read and agreed to the published version of the manuscript.

## Data Availability

The data that support the findings of this study are available on request from the corresponding author. The data are not publicly available due to privacy or ethical restrictions.
